# Nucleus accumbens ghrelin signaling controls anxiety-like behavioral response to acute stress

**DOI:** 10.1186/s12993-024-00244-z

**Published:** 2024-07-04

**Authors:** Leilei Chang, Yecheng He, Tian Tian, Bin Li

**Affiliations:** 1 Women and Children’s Medical Research Center, The First Affiliated Hospital of Nanjing Medical University, Nanjing, 210029 China; 2 Department of Preclinical Medicine, Suzhou Vocational Health College, Suzhou, 215009 China; 3 Department of Neurology, Nanjing Drum Tower Hospital, The Affiliated Hospital of Nanjing University Medical School, Nanjing, 210008 China; 4 Department of Child Health Care, The First Affiliated Hospital of Nanjing Medical University, Nanjing, 210029 China

**Keywords:** Anxiety, Ghrelin, Growth hormone secretagogue receptor, Nucleus accumbens, Acute stress

## Abstract

**Background:**

Anxiety disorders are one of the most common mental disorders. Ghrelin is a critical orexigenic brain-gut peptide that regulates food intake and metabolism. Recently, the ghrelin system has attracted more attention for its crucial roles in psychiatric disorders, including depression and anxiety. However, the underlying neural mechanisms involved have not been fully investigated.

**Methods:**

In the present study, the effect and underlying mechanism of ghrelin signaling in the nucleus accumbens (NAc) core on anxiety-like behaviors were examined in normal and acute stress rats, by using immunofluorescence, qRT-PCR, neuropharmacology, molecular manipulation and behavioral tests.

**Results:**

We reported that injection of ghrelin into the NAc core caused significant anxiolytic effects. Ghrelin receptor growth hormone secretagogue receptor (GHSR) is highly localized and expressed in the NAc core neurons. Antagonism of GHSR blocked the ghrelin-induced anxiolytic effects. Moreover, molecular knockdown of GHSR induced anxiogenic effects. Furthermore, injection of ghrelin or overexpression of GHSR in the NAc core reduced acute restraint stress-induced anxiogenic effects.

**Conclusions:**

This study demonstrates that ghrelin and its receptor GHSR in the NAc core are actively involved in modulating anxiety induced by acute stress, and raises an opportunity to treat anxiety disorders by targeting ghrelin signaling system.

## Introduction

The peptide hormone ghrelin plays an important role in the regulation of feeding and metabolism [1]. Ghrelin is the endogenous ligand of the growth hormone secretagogue receptor (GHSR) [2], which is expressed in numerous brain regions, including the hypothalamus, amygdala and hippocampus [3–6]. However, ghrelin also executes several nonmetabolic functions, such as cognition, reward, stress, depression and anxiety [6, 7]. Intriguingly, the effect of ghrelin system on the anxiety behaviors has attracted more attention. In humans, lower levels of ghrelin are associated with more severe anxiety symptoms in youth with avoidant/restrictive food intake disorders [8]. In animals, Wistar Kyoto rats, which are thought to present more anxiety-like behaviors than Sprague-Dawley and other rats strains, have lower plasma levels of ghrelin than Sprague-Dawley rats [9]. Noticeably, increasing plasma ghrelin levels, through acute calorie restriction or exogenous infusions, have been demonstrated to trigger anxiolytic responses in mice [10–12]. In line with this, ghrelin knockout mice display more anxious after acute restraint stress, compared with wild-type mice [5]. However, different with these findings, several other studies reveal that intracerebroventricular or intraperitoneal administration of ghrelin induces anxiogenic effects [13–16]. The conflicting results indicate that ghrelin system may play a selective role in the regulation of anxiety mediated by distinctive circuits in different brain regions.

A crucial central brain region in the modulation of emotional response is the nucleus accumbens (NAc), which is involved in regulation of anxiety as well as motivation, cognition [17–20]. Neuroanatomically, the NAc has two differently functional sub-structures, the core and the shell [21]. By means of electrical stimulation and neuropharmacology approaches, numerous studies have revealed that the NAc core is strongly implicated in modulating anxiety-like behaviors. High frequency stimulation of the NAc core decreases anxiety level in rodents [22]. Activation of the µ-δ opioid receptor heteromer in the NAc core induces anxiolytic effects [23], while local ablation of glutamate delta-1 receptor from the NAc core produces anxiety-like behaviors [24]. Importantly, deep brain stimulation of the NAc core has been found to decrease ratings of anxiety in patients suffering from treatment-resistant depression [17]. Moreover, using in vivo microdialysis in rats, pre-treatment of ghrelin receptor antagonist significantly decreases the fentanyl-evoked GABA efflux in the NAc and reduces fentanyl-induced behavioral stimulation [25]. Collectively, these studies identify the NAc core as a potential target site for ghrelin’s effect on anxiety.

Here, we investigated the effects of ghrelin signaling in the NAc core on the acute stress-induced anxiety-like behaviors through different approaches. We found that administration of ghrelin into the NAc core produced anxiolytic effects in rats. Ghrelin receptor GHSR is expressed and located in the NAc core neurons. Intra-NAc core infusion of ghrelin reduced the plasma corticosterone and anxiety-like behaviors in responses to acute restraint stress. Molecular upregulation of GHSR in the NAc core ameliorated acute restraint stress-induced anxiety.

## Materials and methods

### Animals

Male Sprague-Dawley rats, aged 7–9 weeks and weighing 220–240 g, were individually housed in a 12-hour light/dark cycle with free access to standard chow and water. All experiments were carried out in accordance with the National Research Council’s Guide for the Care and Use of Laboratory Animals, and approved by the Experimental Animal Care and Use Committee of Nanjing Medical University. All efforts were made to minimize animal discomfort and the numbers of animals used.

### Surgery and microinjection

Rats were anesthetized using sodium pentobarbital (40 mg/kg), placed in a stereotaxic instrument (RWD, China) for brain surgery. Two guide cannulas (length 10 mm, o.d. 0.71 mm, i.d. 0.45 mm) were bilaterally implanted and positioned 2 mm above the NAc core (anteroposterior + 1.3 mm, mediolateral ± 1.5 mm, dorsoventral − 6.5 mm) according to the rat brain atlas [26]. Cannulas were fixed to the skull with dental acrylic cement anchored by stainless steel screws. After surgery, animals were caged individually and allowed to recover for at least 96 h.

For bilateral microinjection in the NAc core, two injection cannulas (length 12 mm, o.d. 0.41 mm, i.d. 0.25 mm) were inserted to protrude 2 mm beyond the tip of the guide cannula. Ghrelin (0.05–0.5 µg; Phoenix Pharmaceuticals, Burlingame, CA), [D-Lys^3^]-GHRP-6 (a selective GHSR antagonist, 5 µg; Tocris, Bristol, UK) or vehicle (0.9% NaCl) were microinjected with Hamilton syringes (0.1 µl each side, lasting 2 min). The effective extent of the drug diffusion in the present study was restricted in the NAc core according to the estimate by extracellular electrophysiological recording units 0.1 to 0.4 mm away from the microinjection site as previous reports [27, 28]. Ghrelin and [D-Lys^3^]-GHRP-6 were dissolved in sterile 0.9% saline. The concentration of drug administration was selected upon previously reported study [15].

### Behavioral assessments

We used the same cohort of animals for the three behavioral tests. The three tests were conducted on the same day in sequence. The time of the interval between the behavioral tests was 10 min.

### Elevated plus maze (EPM) test

The EPM test is one of the most widely used tests for measuring anxiety-like behavior in rodents [29]. The apparatus (made of black PVC) consists of two oppositely positioned open arms (50 × 12 cm) and two oppositely positioned closed arms (50 × 12 × 40 cm) that are intersected at a central square (12 × 12 cm). The maze was elevated above the ground (50 cm). Each rat was placed in the center of the maze, and allowed for 5 min free exploration. The apparatus was cleaned with 75% ethanol after each test to remove olfactory cues. The percent time in the open arms and the percentage of open arm entries were recorded and analyzed using behavioral tracking system TopScan (Clever Sys, Reston, VA). The percentage of open arm entries was calculated as (number of open arm entries/number of total arm entries) × 100.

### Light/dark box (LDB) test

The LDB test takes advantage of a rat’s natural preference for the dark and an aversion to the light, making this a good test of anxiety. The test apparatus consists of a cage divided into two compartments, a light chamber (30 × 30 × 30 cm) and a dark chamber (30 × 30 × 30 cm). The compartments were separated by a partition with an 8 × 8 cm door. Each rat was placed into the middle of the light chamber facing the dark chamber and allowed to move freely between the chambers for 5 min [29]. The apparatus was cleaned with 75% ethanol after each test to remove olfactory cues. The time spent in the light chamber was recorded automatically by behavioral tracking system TopScan (Clever Sys).

### Open field (OF) test

The OF test is a widely used test of anxiety-like behaviors. Animals were individually placed in the center of a chamber (50 × 50 × 50 cm). Spontaneous locomotor activity was monitored by a video camera. The time spent in the center area and total distance traveled during 5 min were examined by behavioral tracking system TopScan (Clever Sys). The apparatus was cleaned with 75% ethanol after each test to remove olfactory cues.

### Quantitative real time PCR (qRT-PCR)

Brains were extracted, placed in cold diethyl pyrocarbonate-PBS, and then sectioned into 1-mm coronal slices in a stainless steel rat brain matrices. Tissue punches corresponding to the NAc core, as confirmed by comparison to the rat brain atlas, were excised using 0.5 mm stainless steel micropunch needles. RNA extraction was performed using TRIzol reagent (Invitrogen, Carlsbad, CA), and reverse-transcribed into cDNA using PrimeScript RT reagent kit (Takara, Japan). cDNA, primers and other reagents were mixed. qRT-PCR was then performed using Fast Start Universal SYBR Green Master (Roche Diagnostics, Indianapolis, IN). The reaction was carried out in the ABI 7500 system (Applied Biosystems, Foster City, CA). For quantification, the quantity of the target gene was expressed relative to the amount of the reference gene (*Gapdh*) to obtain a normalized target expression value. The rationale for primers was selected upon previously reported study [30].

Sequences of primers were as follows: *Ghsr*: forward, 5’-CTA TCC AGC ATG GCC TTC TC-3’ and reverse, 5’-GGA AGC AGA TGG CGA AGT AG-3’; *Gapdh*: forward, 5’-GAA CGG GAA GCT CAC TGG-3’ and reverse, 5’-GCC TGC TTC ACC ACC TTC T-3’.

### Immunofluorescence

Immunofluorescence was performed as previously described [29]. Rats were anesthetized with sodium pentobarbital (65 mg/kg) and perfused transcardially with 100 ml normal saline, followed by 250–300 ml 4% paraformaldehyde in 0.1 M phosphate buffer. Subsequently, the brain was carefully removed, trimmed and post-fixed in the same fixative for 12 h at 4 °C, and then cryoprotected with 30% sucrose for 48 h. Frozen coronal slices (25 μm thick) containing the NAc core were obtained by using a freezing microtome (CM3050S, Leica, Wetzlar, Germany) and mounted on gelatin-coated slides. The slices were rinsed with PBS containing 0.1% Triton X-100 and then incubated in 10% bovine serum in PBS containing 0.1% Triton X-100 for 30 min. Sections were incubated overnight at 4 °C with a rabbit anti-GHSR antibody (1:200; ab95250, Abcam, Cambridge, UK). The specificity of anti-GHSR antibody has been validated in the previous study [31]. After a complete wash in PBS, the sections were incubated in Alexa Fluor 488-conjugated goat anti-rabbit (1:2000; A11008, Invitrogen) for 2 h at room temperature in the dark. The slides were washed and mounted in Fluoromount-G mounting medium (Southern Biotech, Birmingham, AL). Incubations replacing the primary antiserum with control immunoglobulins and/or omitting the primary antiserum were used as negative controls. Images were acquired with a laser scanning confocal microscope (Model SP2 TCS, Leica) and recorded with Leica Application Suite imaging and analysis software (Leica).

### Acute restraint stress

For the exposure to acute restraint stress, rats were restrained in a cylindrical tube for 30 min. The tube (21 cm long, 6.5 cm internal diameter) is made of clear Plexiglas, with sliding plugs to allow fitting of the tube length for the each animal size. The end of the tube had holes allowing free air circulation. Ghrelin or vehicle was administrated 5 min before acute restraint stress. Anxiety-like behavioral tests were performed 5 min after acute restraint stress.

### Plasma corticosterone level analysis

To assess plasma hormone responses to acute restraint stress, blood was collected and centrifuged from the caudal vein immediately after the end of the restraint. A standard corticosterone enzyme-linked immunosorbent assay (ELISA) kit (Abnova) was used to assess plasma corticosterone according to the manufacturer’s instructions. All samples were assayed in duplicate in the same plate.

### Adeno-associated virus (AAV) production and microinjection

The viruses used in our study were purchased from PackGene Biotech (Guangzhou, China). For in vivo knockdown of Ghsr in the NAc core, short hairpin RNA (shRNA) targeting Ghsr mRNA was cloned and packaged into an AAV under the control of a U6 promoter and co-expressing enhanced green fluorescent protein (eGFP) downstream of the U6 promoter (viral titer = 1.39 × 10^13^ vg/ml). The sequence of the GHSR shRNA was CGACTCACTGCCTGACGAA. A scrambled shRNA expressing eGFP was used as a control.

To upregulate the expression of Ghsr in the NAc core, the coding regions of the GHSR was amplified and integrated into the vector (hSyn-EGFP-P2A-Ghsr-3xFLAG-WPRE). The viral titer was 1.15 × 10^13^ vg/ml.

The AAVs were stereotaxically microinjected (0.1 µl) into the bilateral NAc core for knockdown or overexpression of GHSR. The rats treated with AAVs were caged individually. Three weeks were allowed for viral expression before behavioral tests. The knockdown or upregulation of expression of GHSR in the NAc core was assessed by qRT-PCR.

### Histological identification

The animals were anaesthetized with an overdose of sodium pentobarbital after behavioral tests. The brains were collected and submerged in paraformaldehyde. Seven days later, frozen coronal Sect. (80 μm) were prepared, and the microinjection sites were identified according to the rat brain atlas [26]. Data from rats in which the microinjection sites were deviated from the target nuclei were excluded.

### Statistical analysis

All statistical analyses were performed using SPSS 17.0. Shapiro-Wilk test was used for testing the normality of data. All data passed normality test and presented as mean ± SD. Two-tailed unpaired *t*-test, and one-way analysis of variance (ANOVA) with Newman-Keuls post hoc tests were used to determine statistical significance. *P* < 0.05 was considered statistically significant.

## Results

### Intra-NAc core microinjection of ghrelin produces anxiolytic responses

**Fig. 1:**
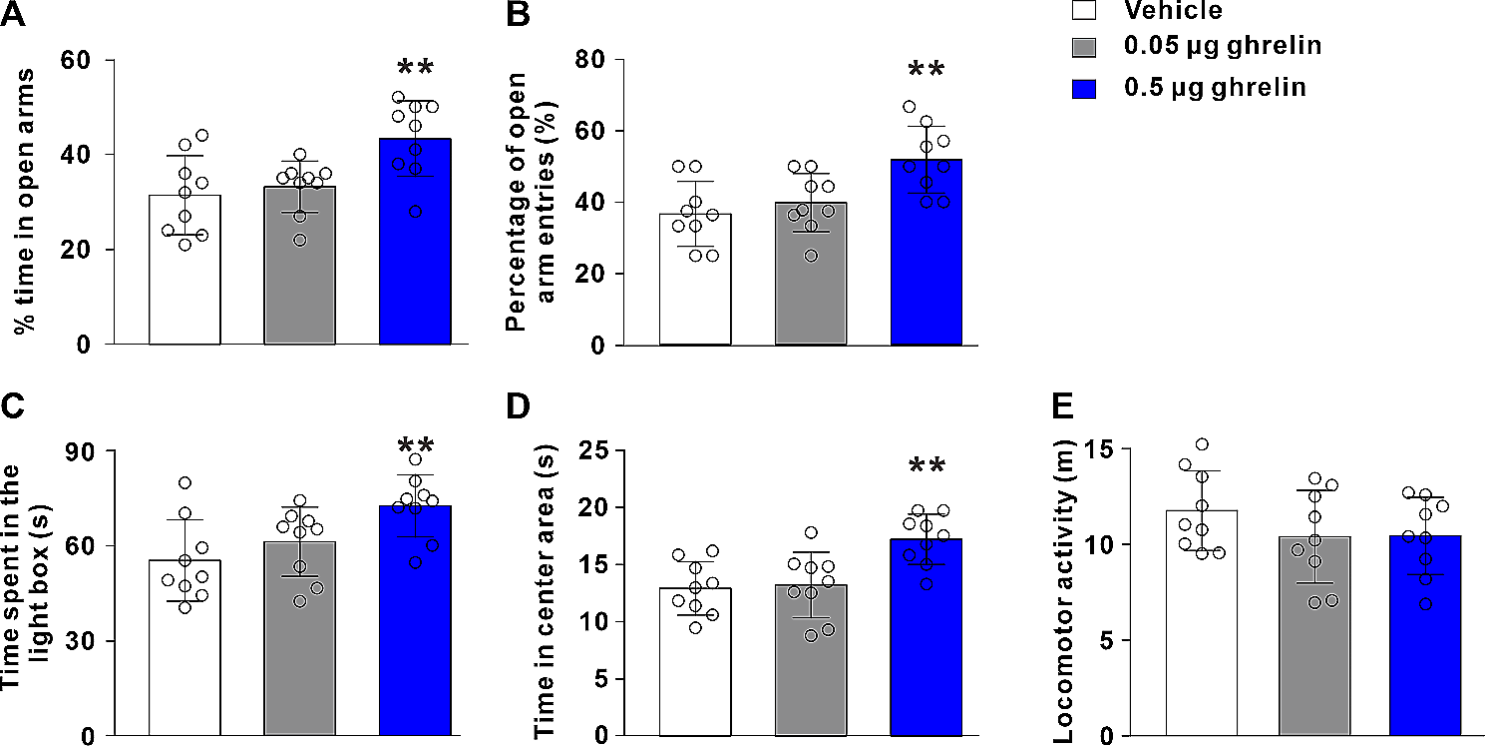
Microinjection of ghrelin into the NAc core elicits anxiolytic effects. **A** and **B** The % time in open arms and percentage of open arm entries of rats with bilateral NAc core infusion of vehicle, 0.05–0.5 µg ghrelin in the EPM test. **C** The time spent in the light box of rats with bilateral NAc core infusion of vehicle, 0.05–0.5 µg ghrelin in the LDB test. **D** and **E** The time in center area and locomotor activity of rats treated by vehicle, 0.05–0.5 µg ghrelin in the OF test. Data are shown as means ± SD; ** *P* < 0.01 compared with the vehicle control group

To determine the effect of acute ghrelin administration on anxiety-like behaviors, rats were exposed to EPM, LDB and OF tests after intra-NAc core ghrelin administration. In the EPM test, there was a significantly main effect of treatment on the % time in open arms and the percentage of open arm entries (F_2, 24_ = 6.90, *P* < 0.01; F_2, 24_ = 7.33, *P* < 0.01). Post hoc analysis showed that bilateral infusion of ghrelin, at dose of 0.5 µg, into the NAc core remarkably increased both the % time in open arms and open arm entries compared with vehicle controls (*P* < 0.01; *P* < 0.01; Fig. 1A-B).

In the LDB test, the ANOVA indicated that an effect of treatment was evident on the time spent in the light box (F_2, 24_ = 5.46, *P* < 0.05). Post hoc test revealed that 0.5 µg ghrelin increased light box time compared with vehicle controls (*P* < 0.01; Fig. 1C).

In the OF test, there was also a remarkable effect of treatment on the time spent in the center of the OF (F_2, 24_ = 8.36, *P* < 0.01). Post hoc analysis showed that 0.5 µg ghrelin increased center time (*P* < 0.01; Fig. 1D). In addition, we did not observe any alterations in the locomotor activity (F_2, 24_ = 1.13, *P* > 0.05; Fig. 1E), suggesting that the ghrelin did not impact locomotor functions. Overall, these results demonstrated that ghrelin microinjection into the NAc core elicited anxiolytic effects.

### GHSR is distributed in the NAc core and GHSR antagonist blocks anxiolytic effects induced by ghrelin

**Fig. 2:**
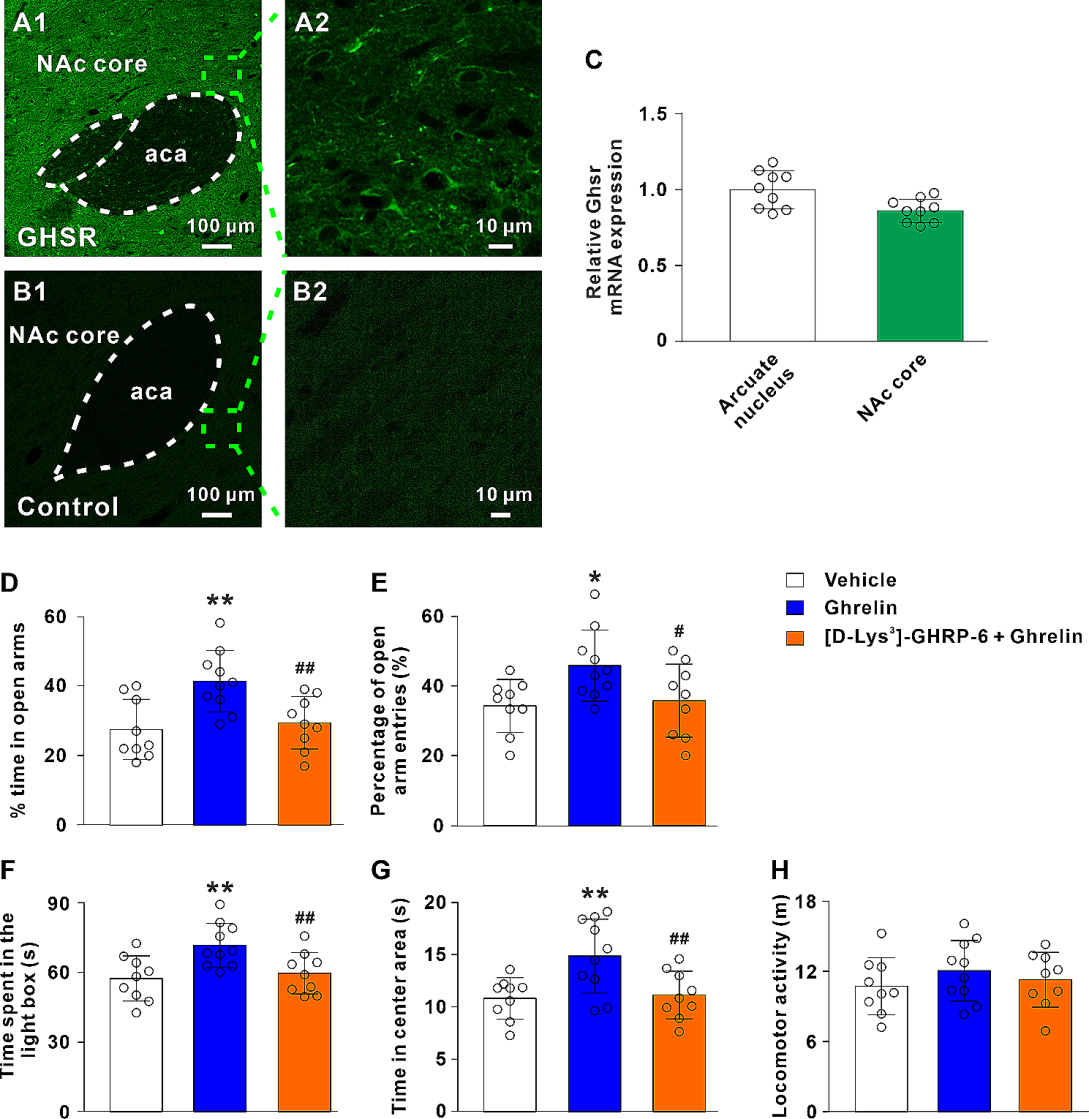
Ghrelin receptor GHSR is distributed in the rat NAc core neurons and blockade of GHSR reverses the ghrelin-induced anxiolytic effects. **A1**-**B2** Immunofluorescence results revealed that GHSR is present in the rat NAc core neurons. **C** The relative expression of GHSR in the arcuate nucleus and NAc core. **D** and **E** The % time in open arms and percentage of open arm entries of rats treated by vehicle, ghrelin or [D-Lys^3^]-GHRP-6 + ghrelin in the EPM test. **F** The time spent in the light box of rats with bilateral microinjection of vehicle, ghrelin or [D-Lys^3^]-GHRP-6 + ghrelin in the LDB test. **G** and **H** The time in center area and locomotor activity of rats with bilateral microinjection of vehicle, ghrelin or [D-Lys^3^]-GHRP-6 + ghrelin in the OF test. Data are shown as means ± SD; * *P* < 0.05, ** *P* < 0.01 for the comparison with the vehicle control group; # *P* < 0.05, ## *P* < 0.01 compared with the ghrelin group. aca, anterior commissure, anterior part

Firstly, we observed the distribution and expression of ghrelin receptor GHSR in the NAc core. The results from immunofluorescence demonstrated that GHSR was distributed in the NAc core neurons in rats (Fig. 2A1-B2). Consistent with the immunofluorescence results, the qRT-PCR results revealed that GHSR expression in the NAc core was slightly less than that in the arcuate nucleus, where GHSR is highly expressed, indicating a high expression of GHSR in the NAc core (Fig. 2C).

Next, we assessed the role of ghrelin receptor GHSR in the anxiolytic behavioral effects of NAc core ghrelin administration. The ANOVA demonstrated a significantly main effect for treatment on the % time in open arms (F_2, 25_ = 7.75, *P* < 0.01; Fig. 2D), the percentage of open arm entries (F_2, 25_ = 4.17, *P* < 0.05; Fig. 2E), the time spent in the light box (F_2, 25_ = 6.46, *P* < 0.01; Fig. 2F) and the center time (F_2, 25_ = 6.61, *P* < 0.01; Fig. 2G). Meanwhile, there was no significant difference in the locomotor function (F_2, 25_ = 0.69, *P* > 0.05; Fig. 2H). Post hoc analysis showed that GHSR antagonist [D-Lys^3^]-GHRP-6 pretreatment blocked the anxiolytic effects caused by NAc core ghrelin administration (Fig. 2D-G). Thus, the above results suggested that the anxiolytic effects of ghrelin were mediated by the GHSR in the NAc core and GHSR was actively involved in the modulation of anxiety-like behaviors.

### Knockdown of GHSR in the NAc core increases anxiety-like behaviors

**Fig. 3:**
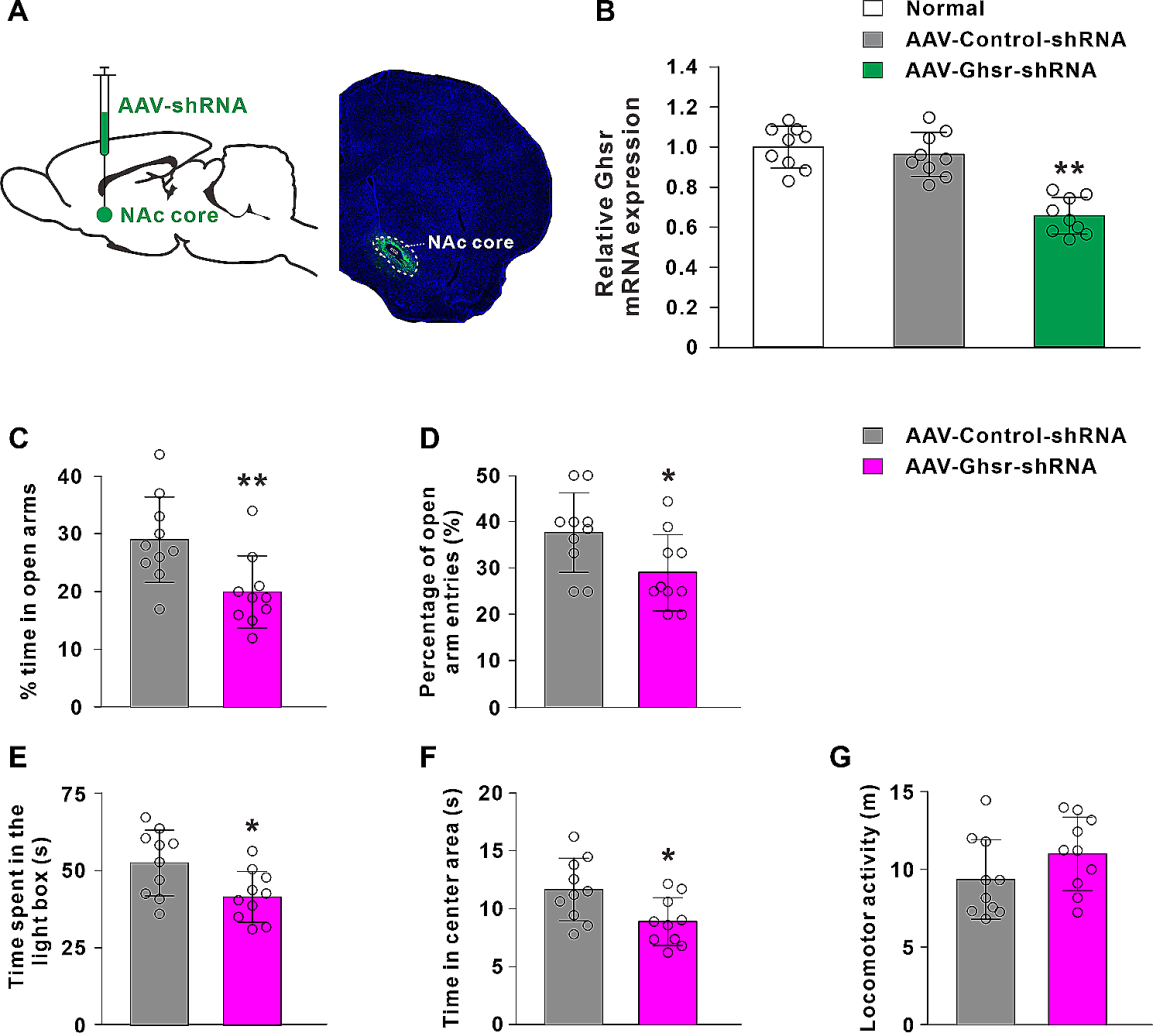
Knockdown of GHSR in the NAc core induces anxiogenic effects. **A** A sagittal brain section showing the microinjection site of AAV in the NAc core and a coronal section showing AAV (eGFP-positive) expression in the NAc core. **B** The knockdown efficiency of GHSR mRNA was evaluated by qRT-PCR. **C**-**G** Knockdown of GHSR in the NAc core decreased the % time in open arms, the percentage of open arm entries, the time spent in the light box and the time in center area, without affecting locomotor activity. Data are shown as means ± SD; * *P* < 0.05, ** *P* < 0.01 for the comparison with the AAV-control-shRNA group

To determine the endogenous ghrelin signaling system in the regulation of anxiety, we generated AAV vectors to knockdown the expression of GHSR in the NAc core. The extent of AAV infection in the NAc core was identified by detecting a fluorescent tag eGFP co-expressed with shRNA (Fig. 3A). The effectiveness of knockdown was evaluated by qRT-PCR. AAV-Ghsr-shRNA remarkably reduced the level of GHSR mRNAs to 65.76 ± 9.18% (*P* < 0.01; Fig. 3B). The behavioral results showed that GHSR knockdown in the NAc core significantly decreased the % time in open arms (t_(18)_ = 2.95, *P* < 0.01; Fig. 3C), the percentage of open arm entries (t_(18)_ = 2.57, *P* < 0.05; Fig. 3D), the time spent in the light box (t_(18)_ = 2.59, *P* < 0.05; Fig. 3E) and the center time (t_(18)_ = 2.57, *P* < 0.05; Fig. 3F). Knockdown of GHSR did not influence locomotor activity (t_(18)_ = 1.49, *P* > 0.05; Fig. 3G). These results demonstrated that knockdown of GHSR in the NAc core was sufficient to induce anxiogenic effects in rats.

### Ghrelin infusion in the NAc core decreases the plasma corticosterone and anxiety-like behaviors induced by the acute restraint stress

**Fig. 4:**
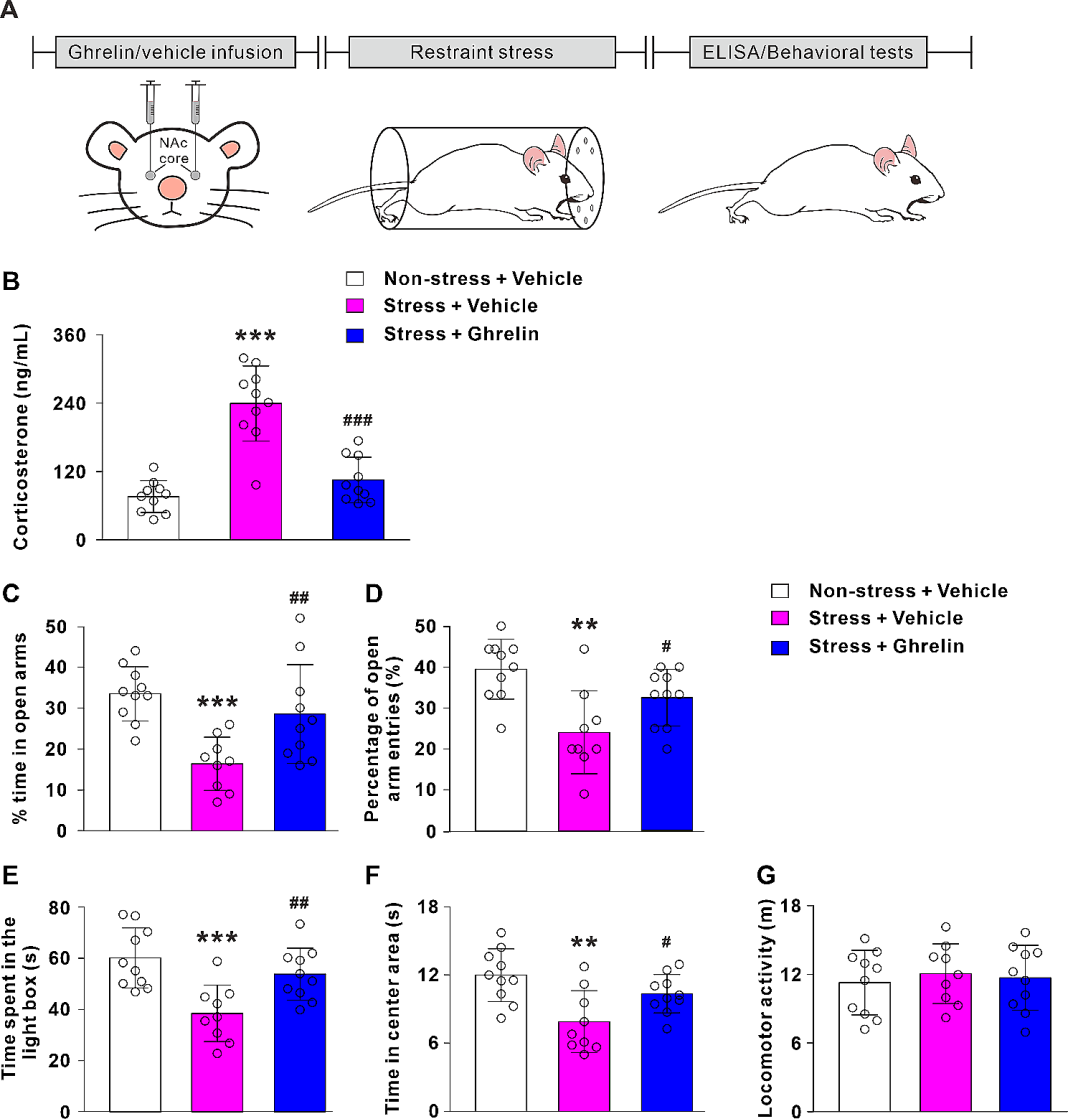
Microinjection of ghrelin into the NAc core reduces acute restraint stress-induced anxiety-like behaviors. **A** Scheme of experimental paradigm showed the acute restraint stress induced anxiety-like behaviors in rats. **B** ELISA analyses showed lower plasma corticosterone level after NAc core ghrelin administration in acute restraint stress conditions. **C**-**G** Acute restraint stress exposure decreased the % time in open arms, the percentage of open arm entries, the time spent in the light box and the time in center area. NAc core ghrelin infusion increased the % time in open arms, the percentage of open arm entries, the time spent in the light box and the time in center area of acute restraint stress rats, without affecting locomotor activity. Data are shown as means ± SD; ** *P* < 0.01, *** *P* < 0.001 for the comparison with the non-stress + vehicle group. # *P* < 0.05, ## *P* < 0.01, ### *P* < 0.001 for the comparison with the stress + vehicle group

In rats, acute restraint stress exposure elicits anxiety-related behaviors [32]. Then, we observed the role of NAc core ghrelin signaling in the stress conditions (Fig. 4A). We examined the corticosterone levels and assessed the effect of NAc core ghrelin microinjection on the activity of HPA axis. It is found that the corticosterone levels of stress animals was significantly increased after exposed to acute restraint stress (*P* < 0.001; Fig. 4B). It is noteworthy that microinjection of ghrelin into the NAc core suppressed the increased levels of corticosterone induced by the acute restraint stress (*P* < 0.001; Fig. 4B).

In the EPM test, the ANOVA analysis revealed that there was a significant treatment in the % time spent in open arms and percentage of open arm entries (F_2, 26_ = 9.19, *P* < 0.01; F_2, 26_ = 8.44, *P* < 0.01). Post hoc test demonstrated that acute restraint stress significantly reduced the % time in opens arms (*P* < 0.001; Fig. 4C) and the percentage of open arm entries (*P* < 0.01; Fig. 4D) compared with non-stress + vehicle group. Microinjection of ghrelin into the NAc core significantly increased the % time in open arms (*P* < 0.01; Fig. 4C) and open arm entries (*P* < 0.05; Fig. 4D) in acute restraint stress rats.

In the LDB test, an effect of treatment (F_2, 26_ = 9.65, *P* < 0.001) was evident on the time spent in the light box. Acute restraint stress treatment decreased the time spent in light box compared with non-stress + vehicle group (*P* < 0.001; Fig. 4E). Ghrelin administration significantly increased the time spent in the light box in acute restraint stress animals (*P* < 0.01; Fig. 4E).

In the OF test, there was an effect of treatment (F_2, 26_ = 7.74, *P* < 0.01) on the time spent in the center area, as acute restraint stress treatment remarkably decreased the center time (*P* < 0.01; Fig. 4F). Ghrelin infusion increased the time in center area of acute restraint stressed rats (*P* < 0.05; Fig. 4F). In addition, treatment did not affect the locomotor activity (F_2, 26_ = 0.19, *P* > 0.05; Fig. 4G). These results revealed that ghrelin infusion in the NAc core reduced anxiety-like behaviors caused by the acute restraint stress.

### Overexpression of GHSR in the NAc core ameliorates acute restraint stress-induced anxiety-like behaviors

**Fig. 5:**
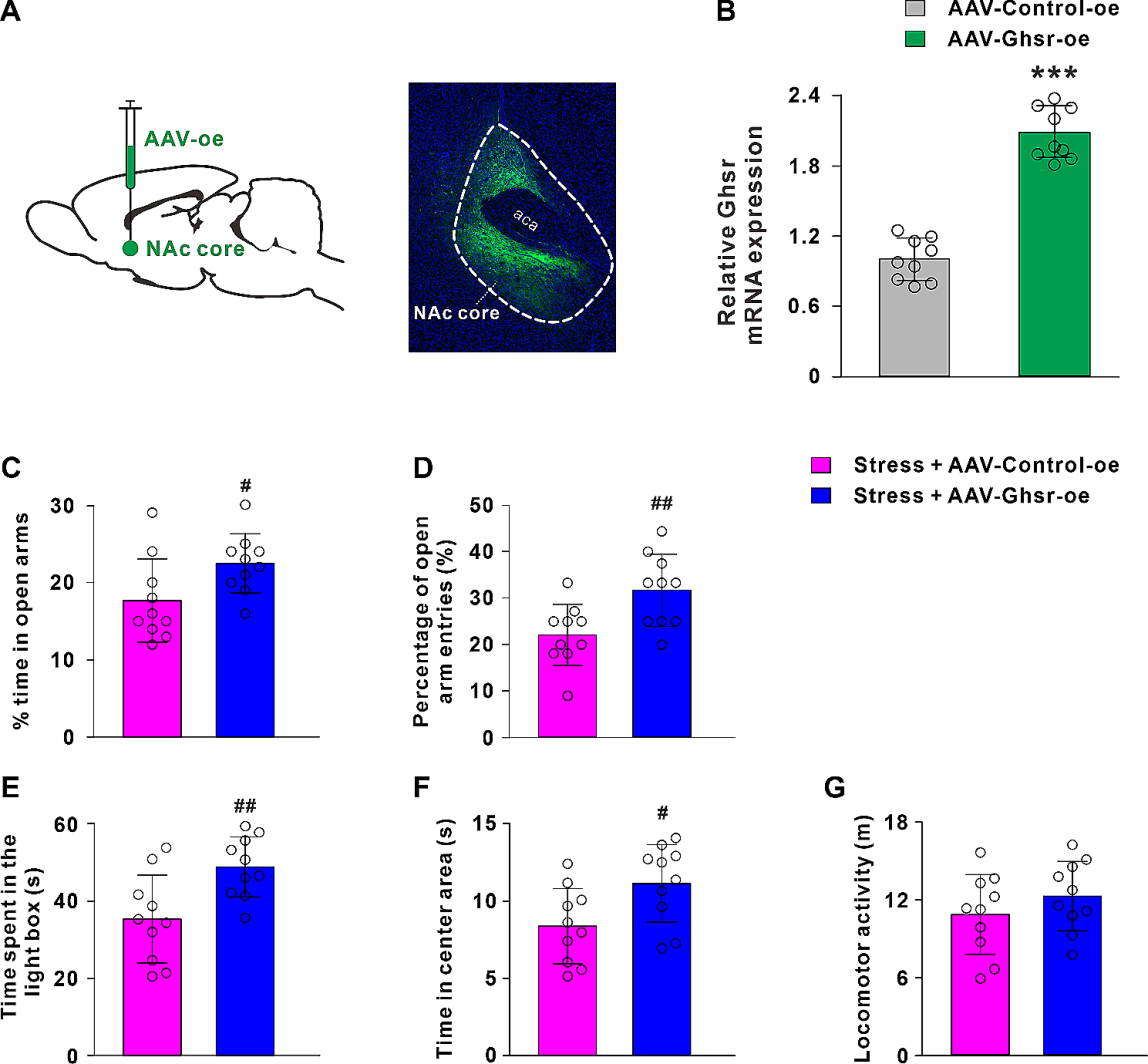
Overexpression of GHSR in the NAc core reverses acute restraint stress-induced anxiogenic effects. **A** A sagittal brain section showing the microinjection site of AAV in the NAc core and a coronal section showing AAV expression in the NAc core. **B** Identification of the overexpression efficiency of GHSR mRNA by qRT-PCR. **C**-**G** Overexpression of GHSR in the NAc core increased the % time in open arms, the percentage of open arm entries, the time spent in the light box and the time in center area of acute restraint stressed rats. Data are shown as means ± SD; *** *P* < 0.001 for the comparison with the AAV-Control-oe group; # *P* < 0.05, ## *P* < 0.01 for the comparison with the stress + AAV-Control-oe group. aca, anterior commissure, anterior part

**Fig. 6:**
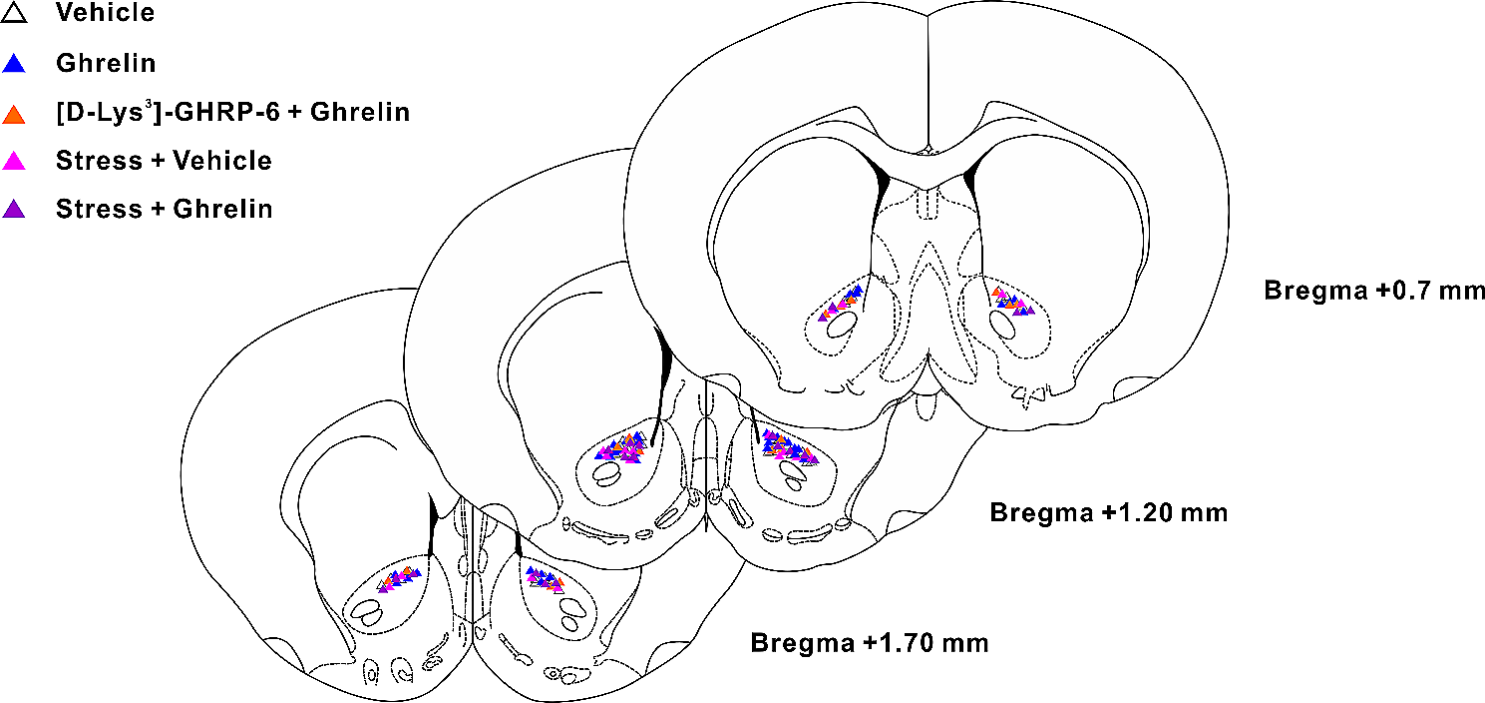
Histological verification of microinjection sites in the NAc core. Histological reconstruction demonstrating the microinjection sites

Furthermore, we utilized an AAV vector to overexpress the GHSR in the NAc core and examined its effect on anxiety-like behaviors caused by acute restraint stress (Fig. 5A). We confirmed the overexpression efficiency of AAV-Ghsr-oe by qRT-PCR. AAV- Ghsr-oe significantly increased the level of GHSR mRNAs to 207.56 ± 22.13% (*P* < 0.001; Fig. 5B). The anxiety-related behavioral results revealed that GHSR overexpression in the NAc core remarkably increased the % time in open arms (t_(18)_ = 2.31, *P* < 0.05; Fig. 5C), the percentage of open arm entries (t_(18)_ = 2.99, *P* < 0.01; Fig. 5D), the time spent in the light box (t_(18)_ = 3.09, *P* < 0.01; Fig. 5E) and the center time (t_(18)_ = 2.49, *P* < 0.05; Fig. 5F) in acute restraint stressed rats. GHSR overexpression had no effect on the locomotor activity (t_(18)_ = 1.10, *P* > 0.05; Fig. 5G). These results suggested that GHSR overexpression in the NAc core was essential to reverse anxiety-like behaviors caused by the acute restraint stress. The histologically identified microinjection sites were reconstructed in Fig. 6.

## Discussion

In the present study, we explored the role of NAc core ghrelin/GHSR signaling system in the acute restraint stress-induced anxiety-like behaviors in rats. We found that administration of ghrelin into the NAc core elicited anxiolytic effects. Ghrelin receptor GHSR is localized and expressed in the neurons of NAc core. Knockdown of NAc core GHSR increased anxiety-like behaviors. Importantly, administration of ghrelin or overexpression of GHSR in the NAc core caused anxiolytic effects in acute stress conditions. Overall, our data indicate that ghrelin signaling in the NAc core actively participate in the modulation of anxiety, and ghrelin/GHSR signaling represents a therapeutic target for novel interventions in anxiety disorders.

The ghrelin signaling system has been suggested to modulate anxiety-like behaviors. However, there is no consensus on whether ghrelin alleviates or aggravates anxiety-like behaviors or anxiety. Increased ghrelin levels in serum, induced by subcutaneous ghrelin administration or caloric restriction, produce anxiolytic effects in the elevated plus maze test in mice [10]. Another study also observes anxiolytic effects after ghrelin infusion to the amygdala [33]. While, some other studies demonstrate that ghrelin exerts anxiogenic effects [14, 15, 34]. These contradictory results could potentially be due to the dosage, study design and timing of administration, the brain site of microinjection in the central nervous system and behavioral tests. In the present study, we focused on the NAc core and observed the effect of ghrelin on the anxiety-like behaviors mediated by the NAc core. The NAc has two major subdivisions, the core and the shell, according to different anatomical connections. It is reported that the NAc core receives input from the prelimbic cortex, dorsal subiculum and projects to the dorsolateral portion of the ventral pallidum. While, the NAc shell receives input from the infralimbic cortex, ventral subiculum and sends projections to the ventromedial portion of the ventral pallidum. In addition to distinctions in their connectivity, the core and shell portions differ in physiological properties, and responses to pharmacological and behavioral manipulations [35]. The NAc core lies at the center of emotional connectivity in the brain, and it is important for regulating anxiety-like behaviors [22, 27]. Our results showed that ghrelin microinjection into the NAc core increased the % time in open arms, the percentage of open entries in the EPM test, the time spent in the light box in the LDB test, and the time in the center area in the OFT test, indicating an anxiolytic effect.

Moreover, ghrelin receptor GHSR is located and expressed in the neurons of the NAc core. Specificity of this effect to GHSR was verified by the fact that co-application of a GHSR antagonist with ghrelin abolished ghrelin’s anxiolytic effects. Moreover, we utilized molecular approaches to examine the role of NAc core ghrelin/GHSR signaling in the anxiety-like behaviors. We found that knockdown of NAc core GHSR decreased the open arm time and entries, light box time, and center time, eliciting anxiogenic effects in rats. This result is consistent with the finding from hippocampus-specific GHSR knockdown in mice [31]. In rats, ghrelin appears to increase extracellular dopamine in the NAc [36], and local intra-NAc injection of ghrelin enhances locomotor activity induced by cocaine [37]. It is possible that ghrelin may target the NAc by acting on GHSR present in neuronal fibers innervating the NAc [38]. The present study focused on GHSR in neurons within the NAc core of rats not on afferents of the NAc core. It is well-known that the primary output of the NAc core is directed to the ventral pallidum, which is a key brain area for the regulation of emotion, motivation, and reward processing [35]. From a neural circuit perspective, ghrelin/GHSR signaling would increase the neuronal activity of the NAc core communicating with the ventral pallidum, and produce anxiolytic responses. Future research studies are required to investigate this issue.

Numerous findings from animal and human studies demonstrate a key involvement of ghrelin in the regulation of stress response [39]. Several studies indicate that ghrelin level in the serum is increased after exposure to psychological stress in both rodents [10, 40] and humans [41, 42]. Next, we determined whether NAc core ghrelin/GHSR signaling regulated anxiety symptoms in a model of acute restraint stress. Previous studies have reported that acute restraint stress induces anxiogenic responses [29, 32]. In line with these studies, we found that exposure of rats to acute restraint stress caused decreased the time and entries in the open arms, the time in the light box and center area, indicating increased anxiogenic responses. Then, our pharmacological results demonstrated that infusion of ghrelin into the NAc core alleviated anxiogenic effects of acute restraint stress. More importantly, our data further reported that increased GHSR signaling in the NAc core was sufficient to ameliorate anxiety-like behaviors in pathological acute stress conditions.

Various studies have revealed that ghrelin present in plasma or cerebrospinal fluid do not acutely reach the central brain region and/or affect reward-related behaviors [43–46]. In any case, ghrelin is not produced in the mouse brain [47], at least not in detectable quantities [48]. Thus, it seems more likely that GHSR in the NAc core plays actions that may depend on its capability to synergize with other G protein coupled receptors to have its anxiolytic effects. It remains to be further clarified in the future study.

Taken together, these results support the view that activation of ghrelin signaling in the NAc core results in anxiolytic effects in basal conditions and protects against the adverse effects of acute stress. There is also a limitation to the current study. All animals we used were male rats. Future studies should also be performed to explore these effects in female rats.

## Conclusions

The current study demonstrates that NAc core ghrelin/GHSR signaling holds a critical role in regulation of anxiety-like behaviors under non-stressed and acute stressed conditions, which raises a potential therapeutic target to treat anxiety disorders.

## Data Availability

No datasets were generated or analysed during the current study.
